# Long-Term Clinical and Biological Outcomes of Biologic Therapy in Severe Asthma: 24-Month Real-World Cohort Study from Romania

**DOI:** 10.3390/jcm15062444

**Published:** 2026-03-23

**Authors:** Corina Mărginean, Andreea Cristina Safta, Dragoș Huțanu, Corina Eugenia Budin, Maria Beatrice Ianosi, Gabriela Jimborean, Edith-Simona Ianosi

**Affiliations:** 1 Oncology and Palliative Care Department, George Emil Palade University of Medicine, Pharmacy, Science and Technology of Târgu Mures, 540139 Targu Mures, Romania; corina.marginean@umfst.ro; 2 Pulmonology Clinic, Mures County Clinical Hospital, 540011 Targu Mures, Romania; ianosi.maria-beatrice@stud18.umfst.ro; 3 Pulmonology Department, George Emil Palade University of Medicine, Pharmacy, Science and Technology of Târgu Mures, 540139 Targu Mures, Romania; dragos.hutanu@umfst.ro (D.H.); gabriela.jimborean@umfst.ro (G.J.); edith.ianosi@umfst.ro (E.-S.I.); 4 Pathophysiology Department, George Emil Palade University of Medicine, Pharmacy, Science and Technology of Târgu Mures, 540139 Targu Mures, Romania; corina.budin@umfst.ro

**Keywords:** severe asthma, biologic therapy, real-world data, remission, fractional exhaled nitric oxide, blood eosinophils, exacerbations

## Abstract

**Background**: Severe asthma remains associated with substantial morbidity despite optimized inhaled therapy. Biologic agents targeting type 2 inflammation improve clinical outcomes; however, real-world evidence regarding the durability of these effects beyond the first treatment year remains limited. The present study extends the follow-up of a previously reported real-world cohort in which 12-month outcomes of biologic therapy were evaluated. **Methods**: We conducted a retrospective observational longitudinal study of adults with severe asthma treated with omalizumab, benralizumab, or dupilumab at a tertiary center in Târgu-Mureș, Romania, between 2020 and 2025, extending follow-up of a previously published real-world cohort. The same patient cohort was followed for an additional period, with longitudinal data collected up to 24 months after biologic therapy initiation. Clinical, functional, and biomarker outcomes were assessed at baseline, 12 months, and 24 months, including Asthma Control Test (ACT) score, forced expiratory volume in one second (FEV_1_% predicted), annual exacerbation rate, blood eosinophil count, and fractional exhaled nitric oxide (FeNO). Remission was defined as clinical (ACT ≥ 20, no severe exacerbations, and no maintenance oral corticosteroids), biological (FeNO < 20 ppb and blood eosinophils < 150/µL), and complete (both clinical and biological). Longitudinal changes were analyzed using the Friedman test with post hoc Wilcoxon signed-rank tests. **Results**: Forty-eight patients were included at baseline, and 41 had available data at 24 months. ACT scores improved from 12 (IQR 11–14) at baseline to 23 (21–25) at 12 months and remained stable at 22 (20–25) at 24 months (*p* < 0.001). Predicted FEV_1_% increased from 50 (39–59) to 78 (68–88) at 12 months and 79 (66–96) at 24 months (*p* < 0.001). Blood eosinophil counts were markedly suppressed, and FeNO levels continued to decrease over time. Exacerbations declined from 2 (2–3) per year at baseline to 0 and 0.5 (0–1) at 12 and 24 months, respectively (*p* < 0.001). At 24 months, clinical, biological, and complete remission were observed in 61.0%, 78.0%, and 41.5% of patients with available paired data, respectively. **Conclusions**: Biologic therapy was associated with sustained clinical and functional improvement over 24 months, accompanied by sustained improvement in type 2 airway inflammation and increasing proportions of patients meeting remission criteria in real-world practice.

## 1. Introduction

Severe asthma is a heterogeneous disease associated with a substantial burden of symptoms, frequent exacerbations, impaired lung function, and increased healthcare utilization. Asthma affects more than 260 million people worldwide and represents a major cause of respiratory morbidity and healthcare utilization. Although most patients achieve adequate disease control with inhaled therapy, approximately 5–10% develop severe asthma requiring advanced treatment strategies. In recent years, increasing attention has focused on type 2 inflammatory pathways and on biomarkers such as blood eosinophils and fractional exhaled nitric oxide (FeNO), which help characterize disease phenotype and guide the selection of biologic therapies targeting specific inflammatory mechanisms. According to international guidelines and major reviews, including those of the Global Initiative for Asthma (GINA) and Brusselle and Koppelman, the introduction of biologic therapies targeting key pathways of type 2 inflammation has markedly improved outcomes in patients with severe asthma [1,2]. These improvements include better asthma control, reduced exacerbation rates, improved lung function, and decreased dependence on oral corticosteroids.

Most of the available evidence regarding the effectiveness of biologic therapy in severe asthma focuses on short- to medium-term outcomes, typically assessed within the first year of treatment [3]. Real-world studies have consistently demonstrated rapid clinical and biological improvements during the first 12 months, with comparable effectiveness across different biologic agents and no clear long-term superiority of one agent over another [4,5]. These findings have supported the growing interest in asthma remission as a meaningful therapeutic goal beyond symptom control alone [3,6,7]. However, definitions of asthma remission remain heterogeneous across studies, and an internationally standardized definition is still evolving.

However, data regarding the durability of these benefits beyond the first year of treatment remain comparatively limited, particularly in real-world settings. While existing evidence suggests that the initial clinical benefits of biologic therapy are generally maintained with long-term use, important uncertainties persist regarding the stability of response over time, the evolution of airway inflammatory control, and the risk of late loss of response [8]. Recent real-world studies have further indicated that response to biologic therapy may be dynamic, with a subset of patients experiencing late failure despite an initially favorable response, underscoring the need for extended longitudinal evaluation beyond the first year of treatment [9].

As an increasing number of patients achieve sustained asthma control or remission under biologic therapy, questions related to long-term disease management, treatment durability, and the clinical significance of maintained remission have become increasingly relevant [10]. In our previously published 12-month retrospective analysis of this real-world cohort, biologic therapy was associated with marked clinical and biological improvement and with substantial proportions of patients achieving remission-related outcomes [11]. However, that study did not address whether these benefits remained stable beyond the first treatment year, whether inflammatory biomarkers continued to evolve over time, or whether remission status changed further with prolonged biologic exposure. Therefore, the present study extends the follow-up of the same cohort to 24 months, focusing specifically on the durability of clinical and biological outcomes beyond the previously reported 12-month analysis. Specifically, we aimed to evaluate longitudinal changes in asthma control, lung function, exacerbation burden, blood eosinophils, and FeNO, as well as the evolution of clinical, biological, and complete remission over time. Given the real-world nature and sample size of the cohort, the purpose of this extension was descriptive rather than predictive, with emphasis on long-term outcome persistence beyond the first year of treatment.

## 2. Materials and Methods

### 2.1. Study Design and Objectives

This study was designed as a retrospective, observational, longitudinal real-world follow-up analysis aimed at evaluating the long-term durability of clinical, functional, and inflammatory responses to biologic therapy in patients with severe asthma. The primary objective was to assess whether improvements and remission-related outcomes observed during the first year of treatment remained stable or evolved further at 24 months following biologic initiation. Secondary objectives included the evaluation of longitudinal changes in asthma control, lung function, inflammatory biomarkers, exacerbation frequency, and oral corticosteroid (OCS) use over the 24-month follow-up period. This analysis represents an extension of a previously reported real-world cohort in which 12-month outcomes were evaluated, expanding follow-up to provide additional information on outcome persistence and remission evolution beyond the first treatment year. The previously published analysis focused on outcomes during the first 12 months of biologic therapy, whereas the present study extends the observation period to 24 months and focuses specifically on durability of response and evolution of remission over time.

### 2.2. Study Population

The study included adult patients with severe asthma treated at the Pulmonology Clinic of the Mureș County Clinical Hospital between 2020 and 2025. Patients were identified through the hospital electronic medical records and the biologic therapy registry of the Pulmonology Clinic. All eligible patients who initiated biologic therapy during the study period were consecutively included in the cohort. A total of 48 patients initiated biologic therapy and were included in the baseline analysis. Among them, 36 patients received benralizumab, 8 omalizumab, and 4 dupilumab at treatment initiation. During follow-up, two patients died (acute myocardial infarction and pneumonia, respectively) and five were lost to follow-up due to discontinuation of routine clinical visits, resulting in 41 patients with available data at 24 months. All analyses were conducted using an available-case approach, without data imputation. No imputation methods were applied due to the observational real-world design. Clinical, functional, and laboratory data were retrospectively extracted from routine follow-up visits documented in the hospital electronic medical records.

The study protocol was approved by the local Ethics Committee (approval no. 937/23 January 2025).

### 2.3. Eligibility Criteria

#### 2.3.1. Inclusion Criteria

Inclusion criteria were: confirmed diagnosis of severe asthma according to GINA recommendations and national reimbursement criteria; eligibility for biologic therapy based on predefined clinical and laboratory thresholds, including at least two severe exacerbations requiring systemic corticosteroids in the preceding year despite high-dose inhaled corticosteroids (ICS) plus long-acting β_2_-agonists (LABA), blood eosinophil count ≥ 300 cells/µL for anti–IL-5/IL-5 receptor therapies, or elevated total IgE levels with documented allergic sensitization for anti-IgE therapy; initiation of treatment with omalizumab, benralizumab, or dupilumab; and availability of clinical, functional, and biomarker data at baseline and at least one follow-up assessment at 12 or 24 months.

#### 2.3.2. Exclusion Criteria

Exclusion criteria included patients who did not meet eligibility criteria for biologic therapy, patients with incomplete baseline data preventing the assessment of remission outcomes, and patients who discontinued biologic therapy before 12 months for reasons unrelated to treatment efficacy.

### 2.4. Background Asthma Treatment

Before starting a biologic, all patients received high-dose inhaled corticosteroid/long-acting β_2_-agonist combinations in accordance with GINA step 5 recommendations. As clinically indicated, additional controller therapies such as theophylline, leukotriene receptor antagonists (LTRA), and long-acting muscarinic antagonists (LAMA) were administered. For partial symptom control, a subset of patients needed maintenance or intermittent oral corticosteroid therapy. Biologic therapies became available in Romania following national regulatory approval: omalizumab since 2008, benralizumab since 2021, and dupilumab since 2022.

### 2.5. Phenotyping and Biologic Selection

Clinical features and type 2 inflammatory biomarkers, such as blood eosinophil counts, fractional exhaled nitric oxide (FeNO), and total and specific IgE levels, were used to phenotype patients before biologic initiation. Patients with eosinophilic asthma were given preference for anti-IL-5/IL-5 receptor therapy, those with allergic asthma were given anti-IgE therapy, and those without a dominant eosinophilic or allergic phenotype were given anti-IL-4/IL-13 therapy. Treatment choices were based on national reimbursement criteria and routine clinical decision-making.

### 2.6. Outcome Measures

#### 2.6.1. Clinical Outcomes

Asthma control was assessed using the Asthma Control Test (ACT) at baseline, 12 months, and 24 months. Exacerbations were defined as worsening of asthma requiring systemic corticosteroids for ≥3 days. Lung function was evaluated using spirometry performed according to international spirometry standards, with forced expiratory volume in one second (FEV_1_) expressed as percent predicted. These outcomes were selected because they represent widely used indicators of treatment response in severe asthma and are commonly reported in real-world studies evaluating biologic therapy effectiveness.

#### 2.6.2. Inflammatory Biomarkers

Inflammatory activity was assessed using blood eosinophil counts and FeNO measurements. A FeNO value < 20 ppb and blood eosinophils < 150 cells/µL were considered indicative of biological remission.

### 2.7. Definition of Remission

Clinical remission was defined as an ACT score ≥ 20, absence of severe exacerbations and no maintenance OCS.

Biological remission was defined as FeNO < 20 ppb and blood eosinophil count < 150 cells/µL.

Complete remission required the concomitant fulfillment of both clinical and biological remission criteria.

Remission status was assessed at 12 and 24 months.

These remission definitions were based on pragmatic criteria used in real-world severe asthma studies and emerging proposals for clinical remission in asthma.

### 2.8. Statistical Analysis

Statistical analysis was performed to evaluate longitudinal changes in clinical outcomes, lung function parameters, inflammatory biomarkers, and remission status over the 24-month follow-up period. Statistical analyses were performed using IBM SPSS Statistics version 26.0 (IBM Corp., Armonk, NY, USA). Normality of continuous variables was assessed using histograms, Q–Q plots, and the Shapiro–Wilk test, confirming non-normal distributions. Continuous variables are reported as median (interquartile range), and categorical variables as frequencies and percentages.

Longitudinal changes across baseline, 12 months, and 24 months were analyzed using the Friedman test, with post hoc pairwise comparisons performed using the Wilcoxon signed-rank test. Comparisons between biologic therapies were conducted using the Kruskal–Wallis test. Changes in categorical outcomes, including remission status, were analyzed using the McNemar test. A two-sided *p*-value < 0.05 was considered statistically significant. Due to small subgroup sizes, biologic-specific analyses were considered descriptive.

Given the limited sample size, attrition at 24 months, and marked imbalance across biologic groups, the study was not designed or powered for multivariable longitudinal modeling, trajectory analysis, or robust predictor analysis. Accordingly, the statistical approach was intended primarily to describe longitudinal outcome patterns in a real-world cohort rather than to establish independent predictors of sustained remission or comparative effectiveness across biologic classes. Post hoc pairwise comparisons were interpreted as exploratory analyses, and results should therefore be interpreted cautiously given the potential risk of type I error due to multiple testing.

### 2.9. Handling Missing Data

Longitudinal analyses were primarily performed using an available-case approach at each time point. For remission comparisons between 12 and 24 months, paired analyses were restricted to patients with available remission data at both time points (*n* = 41). To assess the potential impact of attrition, a sensitivity analysis using a conservative non-responder assumption was additionally performed, in which all patients without 24-month follow-up were considered non-responders and the baseline cohort (*n* = 48) was used as the denominator.

## 3. Results

### 3.1. Study Population and Follow-Up

Of the 48 patients included at baseline, 41 had available data at 24 months. Two patients died during follow-up—one due to acute myocardial infarction and one due to pneumonia—both considered unrelated to biologic therapy. Five patients were lost to follow-up due to discontinuation of routine clinical visits, resulting in incomplete longitudinal data. Patients were followed for up to 24 months after biologic therapy initiation (median follow-up: 24, IQR 23–25 months). Consequently, 41 patients completed the 24-month follow-up and were included in the available-case analysis for long-term outcomes (Table 1).

Patient retention remained complete at 12 months, while attrition occurred between 12 and 24 months, resulting in 41 patients with available long-term follow-up data.

As not all variables were available for all patients at each follow-up time point, all analyses were performed using an available-case approach, without data imputation.

Paired remission analysis included patients with available data at both 12 and 24 months (*n* = 41).

### 3.2. Asthma Control

Asthma control, assessed using the Asthma Control Test (ACT), changed significantly over time (Table 2). A Friedman test demonstrated a statistically significant overall difference in ACT scores across baseline, 12 months, and 24 months of biologic therapy (*p* < 0.001).

ACT scores increased markedly after treatment initiation, reaching peak values at 12 months, and remained significantly improved at 24 months compared with baseline.

Pairwise comparisons using the Wilcoxon signed-rank test showed a significant improvement from baseline to 12 months (*p* < 0.001) and from baseline to 24 months (*p* < 0.001). No statistically significant difference was observed between 12 and 24 months (*p* = 0.068).

### 3.3. Pulmonary Function

Pulmonary function, evaluated by forced expiratory volume in one second (FEV_1_% predicted), showed a significant change over time (Table 3). A Friedman test demonstrated a statistically significant overall difference in FEV_1_% across the three time points (*p* < 0.001).

FEV_1_% improved significantly during the first year of biologic therapy and remained stable at 24 months. Wilcoxon signed-rank tests confirmed a significant increase from baseline to 12 months (*p* < 0.001) and from baseline to 24 months (*p* < 0.001), with no significant difference between 12 and 24 months (*p* = 0.542).

### 3.4. Inflammatory Biomarkers

#### 3.4.1. Blood Eosinophils

Blood eosinophil counts decreased significantly following the initiation of biologic therapy (Table 4). A Friedman test revealed a statistically significant overall difference across baseline, 12 months, and 24 months (*p* < 0.001).

Pairwise comparisons demonstrated a significant reduction from baseline to 12 months (*p* < 0.001) and from baseline to 24 months (*p* < 0.001), with no significant difference between 12 and 24 months, indicating sustained suppression of eosinophilic inflammation.

#### 3.4.2. Fractional Exhaled Nitric Oxide (FeNO)

FeNO levels also changed significantly over time. A Friedman test showed a statistically significant overall difference across the three time points (*p* < 0.001).

Wilcoxon tests demonstrated a significant decrease from baseline to 12 months (*p* < 0.001) and from baseline to 24 months (*p* < 0.001). A further significant reduction between 12 and 24 months (*p* = 0.031) was observed (Table 5).

### 3.5. Asthma Exacerbations

The number of asthma exacerbations changed significantly over time (Table 6). A Friedman test demonstrated a statistically significant reduction across baseline, 12 months, and 24 months (*p* < 0.001).

Wilcoxon tests showed a significant reduction from baseline to 12 months (*p* < 0.001). A statistically significant difference was also observed between 12 and 24 months (*p* = 0.008), reflecting the occurrence of isolated exacerbations in a subset of patients during the second year of treatment. Overall, exacerbation frequency remained markedly lower at 24 months compared with baseline.

### 3.6. Comparison Between Biologic Agents

Changes in asthma control, expressed as ΔACT, did not show statistically significant differences between biologic therapies. However, given the marked imbalance in subgroup sizes, these analyses should be interpreted as exploratory and descriptive rather than comparative. A Kruskal–Wallis test showed no statistically significant differences among treatment groups (*p* > 0.05). Given the small subgroup sizes, biologic-specific comparisons were considered exploratory and descriptive.

### 3.7. Remission Outcomes

Remission status was assessed at 12 and 24 months in patients with available paired data (*n* = 41).

At 24 months, clinical remission was observed in 61.0% of patients, biological remission in 78.0%, and complete remission in 41.5% of patients with available paired data (Table 7).

Using the McNemar test, a statistically significant increase in biological remission rates was observed between 12 and 24 months (*p* < 0.05). Clinical remission rates remained stable, while the proportion of patients achieving complete remission increased at 24 months compared with 12 months.

To test the robustness of our findings, a sensitivity analysis was performed using non-responder imputation (NRI), where all 7 patients missing at 24 months were treated as failures. In this model (*n* = 48), the 24-month rates were: clinical remission 52.1% (25/48), biological remission 66.7% (32/48), and complete remission 35.4% (17/48). Remission proportions remained clinically meaningful in the NRI model.

## 4. Discussion

The present real-world follow-up study evaluated the durability of clinical, functional, and inflammatory outcomes in patients with severe asthma receiving biologic therapy over a 24-month period. The main findings indicate sustained improvements in asthma control, lung function, exacerbation burden, and inflammatory biomarkers, together with increasing proportions of patients meeting remission criteria over time.

While our previous retrospective analysis demonstrated that biologic therapy can induce clinical and biological remission within the first year of treatment, the present study extends these findings by evaluating the durability and longitudinal evolution of outcomes over a 24-month follow-up period, representing one of the limited real-world longitudinal extensions beyond the first treatment year. In the earlier 12-month analysis of this cohort, biologic therapy was associated with rapid and clinically meaningful improvements in asthma control, lung function, and inflammatory biomarkers [11,12]. The current follow-up suggests that these benefits appear largely maintained over time.

Importantly, clinical and functional outcomes showed sustained stability during the second year of biologic therapy. Asthma control, assessed by ACT scores, remained significantly improved at 24 months compared with baseline, without a significant decline between 12 and 24 months, indicating durable symptom control under continued treatment. Similarly, lung function improvements, reflected by increases in FEV_1_% predicted, were achieved primarily during the first year and subsequently maintained, suggesting that early functional gains persist over longer periods of treatment. However, interpretation should consider the available-case design, which may overestimate long-term persistence among treatment responders. These findings are in line with real-world evidence indicating long-term durability of clinical response to biologic therapies in severe asthma [8].

One of the relevant findings of this extended follow-up concerns the sustained suppression of type 2 inflammatory biomarkers. Blood eosinophil counts showed a rapid and profound reduction following biologic initiation and remained stable over the 24-month period, while FeNO levels showed a further modest decline during the second year of therapy, although the clinical significance of this incremental reduction remains uncertain. However, interpretation of biomarker-defined remission requires caution, particularly in cohorts predominantly treated with anti–IL-5/IL-5 receptor agents such as benralizumab. In these therapies, suppression of circulating eosinophils represents a direct pharmacodynamic effect and may therefore partly reflect treatment-related biomarker modulation rather than complete disease modification. Consequently, biomarker-based remission definitions should be interpreted in the context of biologic mechanism of action, and pooled analyses across biologic classes may have limited mechanistic interpretability [13,14].

Exacerbation frequency decreased markedly after treatment initiation and remained substantially lower at 24 months compared with baseline. Although isolated exacerbations occurred in a small subset of patients during the second year, the overall exacerbation burden remained minimal. This pattern is consistent with long-term observational data showing that while complete elimination of exacerbations is not universal, biologic therapy appears to confer sustained protection against severe disease worsening over time [15].

A central objective of the present study was to evaluate the evolution of remission outcomes over time. At 24 months, more than half of patients achieved clinical remission, nearly three-quarters achieved biological remission, and ~42% fulfilled criteria for complete remission. Notably, biological remission increased significantly between 12 and 24 months, whereas clinical remission remained stable. Complete remission showed a numerical increase over time, although this did not reach statistical significance. These findings suggest that some remission domains may continue to evolve over time in severe asthma, although remission definitions remain heterogeneous across studies and may be influenced by biomarker selection and treatment mechanisms.

When contextualized within the existing literature, our remission rates compare favorably with large registry-based studies. In the Danish Severe Asthma Register, clinical remission was reported in approximately 19% of patients after 12 months of biologic therapy using a stringent composite definition [16]. Similarly, long-term real-world analyses have shown complete response rates ranging between 27% and 35% after extended follow-up, depending on the criteria applied [17]. The higher remission rates observed in our cohort at 24 months support the concept that remission outcomes may continue to evolve beyond the first year of treatment in some patients, suggesting that longer follow-up may provide additional insight into long-term response patterns.

Registry data further emphasize the heterogeneity of response to biologic therapy. Analyses from the International Severe Asthma Registry demonstrated that although many patients achieve clinically meaningful responses, approximately 40–50% do not meet predefined response or super-response criteria [18]. These observations align with our findings and underscore the need for individualized treatment goals and cautious interpretation of remission outcomes in real-world populations.

Persistent airflow obstruction emerged as a key limiting factor for achieving complete remission. Previous long-term studies have shown that failure to normalize lung function remains one of the most common reasons for incomplete response, even in patients with well-controlled symptoms and suppressed inflammation [19,20]. This finding highlights the importance of early intervention, before irreversible airway remodeling develops, and supports the notion that functional remission may represent the most challenging component of complete disease remission [21]. Given the advanced disease stage in our cohort, structural airway remodeling may have limited the reversibility of functional impairment.

Beyond efficacy, long-term safety and treatment continuation represent critical considerations. Systematic reviews and long-term observational studies have consistently demonstrated a favorable safety profile for biologic therapies, with low rates of treatment-related adverse events and no new safety signals emerging during extended follow-up [13]. These data, together with evidence supporting sustained clinical benefit, support current recommendations to avoid unnecessary discontinuation of biologic therapy in patients who derive long-term benefit [8].

The strengths of the present study include the extended real-world follow-up period; the simultaneous evaluation of clinical, functional, and inflammatory outcomes; and the assessment of remission-related endpoints over a 24-month treatment period. In addition, the study reflects routine clinical practice in a tertiary referral center, providing insight into long-term treatment outcomes in an unselected severe asthma population.

Several limitations of the present study should be acknowledged. The retrospective design and relatively small cohort size limit generalizability. Additionally, loss to follow-up and two deaths during the extended observation period reduced the number of patients available for 24-month analyses. To assess the potential impact of missing data, a sensitivity analysis using a conservative non-responder assumption was performed, in which deceased patients and those lost to follow-up were treated as non-responders. Under this assumption, remission proportions were attenuated, highlighting the influence of missing data in long-term real-world studies. Longitudinal analyses were primarily performed using available cases at each time point, and survivorship bias therefore cannot be excluded, meaning remission rates may be overestimated among patients who remained in follow-up. These limitations are inherent to observational real-world longitudinal research. Additionally, pooling biologic agents with distinct mechanisms and eligibility criteria limits biologic-specific interpretability. Additionally, the marked imbalance in biologic group sizes limits the ability to draw meaningful comparisons between biologic agents, and the absence of statistically significant differences should not be interpreted as evidence of equivalent effectiveness. In our cohort, patients who remained in follow-up generally continued the initially prescribed biologic therapy throughout the observation period, and no systematic switching or planned discontinuation attempts were documented. However, treatment persistence, switching patterns, and discontinuation strategies were not formally analyzed, which may influence long-term outcome interpretation. Comorbidity burden, baseline disease severity, smoking history, BMI, oral corticosteroid dependency, and adherence to background therapy may also have influenced treatment response and remission outcomes. However, the relatively small cohort size and limited number of remission events precluded robust multivariable or subgroup analyses exploring these factors.

In conclusion, this 24-month real-world follow-up study suggests that biologic therapy in severe asthma leads to sustained clinical control, durable lung function improvement, sustained suppression of type 2 inflammatory biomarkers, and increasing rates of remission over time. These findings support the long-term continuation of biologic therapy in appropriately selected patients and emphasize the importance of extended follow-up when evaluating treatment success beyond the first year while highlighting the need for larger prospective longitudinal studies to confirm durability of remission trajectories.

## 5. Conclusions

Biologic therapy was associated with sustained improvements in asthma control, lung function, inflammatory biomarkers, and exacerbation burden over 24 months in this real-world severe asthma cohort. Clinical and functional gains achieved during the first year appeared to be maintained during the second year, while FeNO showed further improvement, although biomarker changes may partially reflect mechanism-driven treatment effects. Remission outcomes evolved over time, with biological remission increasing significantly and complete remission reaching 41.5% at 24 months within the limits of an available-case longitudinal analysis. These findings support the long-term clinical value of biologic therapy in appropriately selected patients and highlight the importance of extended follow-up when evaluating treatment success beyond the first year.

## Figures and Tables

**Table 1:** Patient retention and attrition during follow-up.

Time Point	Overall (*n* = 48)	Benralizumab (*n* = 36)	Omalizumab (*n* = 8)	Dupilumab (*n* = 4)
Baseline	48	36	8	4
12 Months	48	36	8	4
24 Months	41	32	6	3
Deaths	2	1	1	0
Lost to follow-up	5	3	1	1

Data are presented as absolute numbers. *n*, number of patients.

**Table 2:** Evolution of asthma control assessed by ACT during biologic therapy.

Time Point	ACT Score, Median (IQR)	*n*
Baseline	12 (11–14)	48
12 Months	23 (21–25)	48
24 Months	22 (20–25)	41
Friedman Test	*p* < 0.001	

Data are presented as median (interquartile range). *n*, number of patients; ACT, Asthma Control Test. Differences across time points were assessed using the Friedman test.

**Table 3:** Evolution of pulmonary function assessed by FEV_1_% predicted during biologic therapy.

Time Point	FEV_1_% Predicted, Median (IQR)	*n*
Baseline	50 (39–59)	48
12 Months	78 (68–88)	48
24 Months	79 (66–96)	41
Friedman Test	*p* < 0.001	

Data are presented as median (interquartile range). *n*, number of patients; FEV_1_, forced expiratory volume in one second. Differences across time points were assessed using the Friedman test.

**Table 4:** Evolution of blood eosinophil counts during biologic therapy.

Time Point	Blood Eosinophils (Cells/µL), Median (IQR)	*n*
Baseline	735 (432–1073)	48
12 Months	0 (0–95)	48
24 Months	0 (0–153)	41
Friedman Test	*p* < 0.001	

Data are presented as median (interquartile range). *n*, number of patients. Differences across time points were assessed using the Friedman test.

**Table 5:** Evolution of fractional exhaled nitric oxide (FeNO) levels during biologic therapy.

Time Point	FeNO (ppb), Median (IQR)	*n*
Baseline	32.5 (24–45)	48
12 Months	18 (13–21)	48
24 Months	11 (9–18)	41
Friedman Test	*p* < 0.001	

Data are presented as median (interquartile range). *n*, number of patients; FeNO, fractional exhaled nitric oxide. Differences across time points were assessed using the Friedman test.

**Table 6:** Evolution of asthma exacerbation frequency during biologic therapy.

Time Point	Exacerbations/Year, Median (IQR)	*n*
Baseline	2 (2–3)	48
12 Months	0 (0–0)	48
24 Months	0.5 (0–1)	41
Friedman Test	*p* < 0.001	

Data are presented as median (interquartile range). *n*, number of patients. Differences across time points were assessed using the Friedman test.

**Table 7:** Remission outcomes at 12 and 24 months of biologic therapy.

Remission Category	12 Months, *n* (%)	24 Months, *n* (%)	McNemar *p*-Value
Clinical remission	24 (58.5%)	25 (61.0%)	NS
Biological remission	27 (65.9%)	32 (78.0%)	<0.05
Complete remission	14 (34.1%)	17 (41.5%)	NS

Data are presented as number (percentage) of patients with available paired data. *n*, number of patients; NS, not significant. Differences between 12 and 24 months were assessed using the McNemar test.

## Data Availability

The data presented in this study are available on reasonable request from the corresponding author. The data are not publicly available due to the inclusion of sensitive clinical information that could compromise participant privacy.

## Abbreviations

ACT	Asthma Control Test
FEV_1_	Forced Expiratory Volume in One Second
FeNO	Fractional Exhaled Nitric Oxide
ICS	Inhaled Corticosteroids
LABA	Long-Acting β_2_-Agonists
OCS	Oral Corticosteroids
